# Sports-Related Concussion Is a Personalized Issue—Evaluation of Medical Assessment and Subjective Feeling of the Athlete in a German Level 1 Trauma Center

**DOI:** 10.3390/jpm12101596

**Published:** 2022-09-28

**Authors:** Johannes Weber, Lorenz Huber, Borys Frankewycz, Werner Krutsch, Volker Alt, Dominik Szymski

**Affiliations:** 1 Department of Trauma Surgery, University Medical Centre Regensburg, Franz-Josef-Strauss Allee 11, 93053 Regensburg, Germany; 2 FIFA Medical Centre of Excellence, University Medical Centre Regensburg, 93053 Regensburg, Germany; 3 SportDocsFranken, 90473 Nuernberg, Germany

**Keywords:** athletic injury, sports medicine, emergency medicine, head trauma, concussion

## Abstract

Sports-related concussions (SRC) have developed into a highly discussed topic in sports medicine over the last few years and demonstrate a severe issue in the personalized treatment of patients. This retrospective cohort study investigated 86 patients with sports-related concussions in a level 1 trauma center, relating to the mechanism, symptoms, medical history, acute therapy including first assessment and the return to sport. The research is based on medical records as well as questionnaires six months after hospitalization. Loss of consciousness for under 30 min (41.2%), headache (36.5%) and amnesia (29.4%) were the most frequent symptoms when presenting in the emergency room. During the hospitalization, mainly headache and vertigo were documented. Most concussions occurred after incidents in equitation and cycling sports; the most common mechanism was falling to the ground with a subsequent impact (59.3%). At the time of discharge from hospital, in 13.4% of all cases, concussion symptoms were still documented in medical records, in contrast to 39.5% of the concerned athletes who reported symptoms for longer than 24 h, and 41.0% who reported ongoing post-concussion symptoms after six months. Concussions are difficult-to-treat disorders with a challenging diagnostic process and many symptoms in various values and levels of persistence. Therefore, a patient-involving treatment with a complaint-dependent return to sport process should be applied to concerned athletes.

## 1. Introduction

In recent years, sports-related concussions (SRC) have become one of the most thrilling topics in sports medicine [1,2,3]. Historically speaking, the investigation of concussions in sports had already started back in 1928, with the description of neurological deficits in boxers (“punch drunk”) [4,5]. Since at least 2005, with the investigation of the cohesion of chronic traumatic encephalopathy (CTE) in NFL players after repetitive concussions, public interest has grown rapidly [6,7]. Nowadays, and especially in regard to football, heading and the impact on the head are heavily discussed. Prohibitions of headers in youths, which were recently introduced, for example in the USA, have also raised awareness for this subject [8,9]. In addition to soccer, the most common sports in Germany are team sports such as basketball and handball, or individual sports such as running, cycling and weight training. Concussions can thereby be caused by a direct impact on the head, or a transmitted force induced by an impact somewhere else on the body. Neurological impairment, as the main symptom, occurs rapidly and mostly disappears spontaneously after a short period of time. A loss of consciousness might also be part of the symptoms. Structural changes are detected rarely in conventional imaging, but functional MRI (fMRI) has shown relevant changes in brain activity in affected areas. Pathophysiologically, a complex cascade leads to an energy discrepancy with reduced functionality of the brain cells [1,10,11]. After experiencing a traumatic brain injury (TBI), a period of time where athletes are still vulnerable and at a high risk of permanent damage due to repeated concussions was reported [12].

Even though the main part of the evaluation of concussions is obtained in emergency departments, there is still a lack of knowledge and practical skills, and an underdiagnosis of SRC is expected [13,14]. Due to the various number of symptoms and the time of their occurrence, a personalized approach to concussion treatment is required.

Therefore, this study investigates first-time sports-related concussions in a German Level 1 trauma center, focusing on initial and long-term symptoms. The comparison of the first examination and a follow-up questionnaire forms the basis of this research project.

## 2. Materials and Methods

### 2.1. Study Population

The present study retrospectively analyzes sport-associated head concussions in a level 1 trauma department within the year 2018. All athletes aged over 18 and younger than 50 years, who were treated with the diagnosis of a sports-related concussion in our emergency department, were selected for inclusion in the study population. In Germany, patients with a loss of consciousness or neurological issues are generally admitted for inpatient monitoring. After completing a surveillance period in hospital, the athletes were informed about this study and a written consent was obtained to participate in the study. Six months after the incident, a written standardized follow-up questionnaire was sent to the patients to evaluate persistent symptoms, the return to training and sports after the end of hospitalization and to evaluate possible further injuries after discharge. Further medical surveillance was performed by local general physicians directly after discharge from the hospital. Further follow-ups were carried out in personalized time frames by the general physicians. A follow-up investigation in our hospital was only performed in severe cases, 4 to 6 weeks after discharge. Missing consent or missing injury report data were exclusion criteria. The study was approved by the Ethics Committee of the University of Regensburg (no. 15-101-0134).

### 2.2. Data collection

The data collection was inspired by the Berlin Consensus statement of concussions and the multicenter study of Winkler et al. (2016) [2,15]. A standardized questionnaire on basis of the Consensus statement was used for data collection. The first part of the research analyzed the doctor’s letter and the clinical record data of the patient in a standardized pattern. The focus was set on the cause of the injury and the type of sport being practiced during the injury. In addition, occurring symptoms and secondary injuries during the clinical surveillance, as well as diagnostic procedures, were included in the statistics. Six months after the initial trauma, the follow-up questionnaire was sent to the participating athletes. Here, previous concussion history, former injuries to the head and the detailed trauma mechanism were evaluated. Furthermore, the diagnosis placement, interruption of the activity, persistence of complaints, return to activity and knowledge about concussion were evaluated.

### 2.3. Statistical Analysis

Continuous data were expressed as means ± standard deviation (SD) and categorical data as frequency counts (percentages). Odds ratios are reported as effect estimates. The significance level was set to *p* < 0.05. All analyses were performed with IBM SPSS Statistics, version 25.0 (Armonk, NY, USA).

## 3. Results

### 3.1. Analysis of Medical Record

Eighty-six patients, who were treated for sports-related concussion in a trauma level 1 emergency department, were included in this study. During the accident, the injured patients were mainly practicing equitation (24/85, 28.2%), football (22/85, 25.8%) and cycling or mountain biking (MTX, 18/85, 21.2%). 

In total, most athletes sustained their SRC after a fall, with the subsequent impact occurring without any physical contact (45/85, 52.9%; *p* < 0.01). Impact caused by direct physical contact (19/85, 22.4%) and impact caused by a ball or another object (8/85, 9.4%) were further frequent mechanisms. Observing the different types of sports, an accumulation of concussions caused by direct physical contact in ball sports (18/19, 94.7%; *p* < 0.01) attracted attention. Whereas in equitation, the type of sport with the most SRCs, 91.7% of SRCs were reported from a fall with a subsequent impact without physical contact (22/24, 91.7%; *p* < 0.01). Cycling and mountain biking (MTX) were also mainly characterized by falls (12/18, 66.7%; *p* < 0.05) (Table 1).

Relating to the doctor’s letter at the end of the treatment, 13.1% (11/84) of the athletes showed remaining symptoms at the end of the monitoring period. Only one patient needed an extension of observation (1.2%), due to persistent severe symptoms which faded after 48 h surveillance. Radiological imaging, which was made after 24 h, showed no intracerebral bleeding in this case. The mean duration of hospitalization was 38.4 h (Table 2), including patients with applied surgical therapy due to secondary diagnoses, with 76.5% (65/85; *p* < 0.05) of all patients being released within the first day after experiencing an SRC (Table 3). A total of 5.9% (5/85) refused to be monitored in hospital. The level of surveillance ranged between no inpatient monitoring necessary and 5 days. During the treatment, 41.2% (35/85) received radiological head imaging by cranial computer tomography (CCT) or magnetic resonance imaging (cMRI). In particular, in patients with SRC sustained in equitation and cycling, radiological imaging was applied more often than in ball sports (odds ratio: 2.3, 2.7, respectively). Isolated concussions without any additional injuries occurred only in half of the treated patients. Further injuries were mainly contusions of the trunk and the extremities (40%), followed by skin lesions (21.2%). Severe musculoskeletal trauma such as fractures or ligament injuries occurred in 16.5% of the cases. Directly after concussion, athletes mainly reported unconsciousness (*n* = 35), headache (*n* = 31) and retrograde amnesia (*n* = 25) (Table 2).

### 3.2. Medical History and Behavior after Incident

Overall, 43.6% of the athletes indicated having had a previous concussion in their medical history. Of these, 58.8% had already reported more than one concussion. Most of the previous concussions (88.2%) also occurred in sports. Only 59.0% of the athletes who had already experienced an SRC were treated in hospital due to the previous insult. In addition, the medical history of neuropsychological disorders of these patients was also collected (Table 3). Here, only 38.5% declared having sufficient personal knowledge about concussions. Moreover, athletes with a lack of knowledge more often classified concussions as a severe injury (70.8%; *p* < 0.05). 

After the incident, 89.7% of the patients with an SRC interrupted activity, but only 69.2% terminated it for the rest of the day. First assessment of trauma sequelae was made by the athletes or teammates in nearly 50% of cases. In 41.1%, concussion symptoms disappeared within the first four hours after the incident, whereas nearly one third (11/38) reported symptoms lasting for more than three days (Table 3).

### 3.3. Remaining Symptoms after 6 Months and Return to Sports

**Figure 1:**
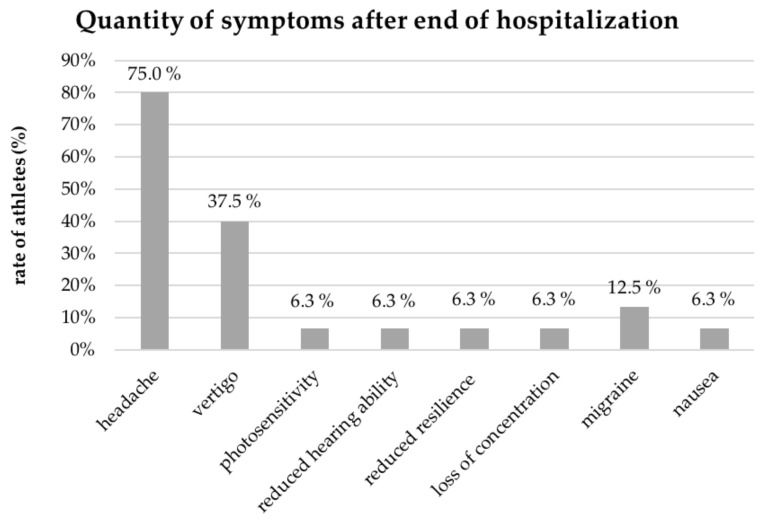
Symptoms after discharge from hospital.

Six months after the SRC, 41.0% of the athletes still reported remaining symptoms caused by the SRC after their discharge from hospital. Women were significantly more likely to report being affected by long-term symptoms (*p* = 0.02) compared to men. The most reported symptoms were headache (75.0%) and nausea (37.5%) (Figure 1). A return to sports at the same level was possible in nearly 90% of all cases, but 24 (61.5%) of the athletes complained about lower activity levels at the six-month follow-up (Table 3).

## 4. Discussion

The present study retrospectively investigated sports-related concussions in a level 1 trauma department in Germany, with its focus on the persistence of symptoms, injury patterns, diagnostics and the return to sport, and thereby turned attention to individual differences and personal perceptions.

The most important result of the examination is the significant difference between the symptoms indicated at the time of discharge from medical treatment in the doctor’s letter and the patient’s questionnaire performed 6 months after discharge. In the evaluation of the medical records, 13.1% of the athletes had indicated symptoms after completing the medical surveillance period, whilst in the questionnaire performed 6 months after an SRC, 41.0% still indicated remaining symptoms in the self-report. Symptoms of a concussion sometimes can be difficult to discover because of their diversity and late onset, up to 48 h after the impact [16], and therefore, are overlooked in clinical settings [13,17,18,19]. Directly after the accident, the most common reported symptoms were the loss of consciousness (41.2%) and headache (36.5%). However, in the patient-reported follow-up investigation after 6 months, the most common described symptoms were headache (75%) and vertigo (37.5%). Often, re-evaluating SRCs’ symptoms is insufficient during the surveillance period by medical personnel. In many cases, no follow-up treatment is applied to the patients, although they need a detailed second or ongoing evaluation with strict guidelines [14,20,21]. In other situations, patients themselves are sometimes not aware of the symptoms due to a lack of knowledge, while others want to enforce a fast discharge and return to work or sport. In particular, successful athletes may take a higher risk to conceal symptoms in order to avoid a longer break from training [1]. Long-term signs of concussions are a major cause of prolonged observation and longer hospitalization [1,14,20,21]. Depending on the type of sport, rates of persistent symptoms between 10 and 30% have been described in the literature. In particular, contact sports and younger athletes showed an increased risk [22,23]. The persistence of symptoms after a concussion is known as post-concussion syndrome (PCS). The World Health Organization did not define a specific time frame in the International Classification of Disease-10 (ICD-10) coding, but the persistence of three or more of the following symptoms—headache, fatigue, dizziness, irritability, insomnia and concentration or memory difficulty—is obligatory [24]. Immediately after the incident, the majority of the athletes in this study reported losing consciousness (41.2%) and headache (36.5%). Although many symptoms faded after hours, nearly one third of the patients had to deal with symptoms for more than 3 days. In detail, headache and neck pain as well as fatigue and drowsiness are described in the literature as the main symptoms of patients with mTBI [25]. Persistent headache is a common symptom after concussion and was noticed in some populations in up to 37% of the cases, 45 days after the injury [25]. The variance of cerebral blood flow is discussed as one of the main mechanisms of PCS. During rest, a reduced blood flow was noticed, whilst during exercise, an excessive increase with an accompanying headache proceeded. Furthermore, abnormal cerebral blood flow was recorded for up to a month after the impact [22]. The high rate of patients with a loss of consciousness as a symptom may be explained by the structure of the study center as university hospital serving as a level 1 trauma center. In addition, a reduced rate of admission of patients when not experiencing a loss of consciousness is possible.

The analyzed patients experienced an SRC mainly after a fall with a subsequent impact, but without any physical contact. This mostly occurred in equitation and cycling sports, whereas direct contact and an impact with a ball or any other object was the key injury mechanism in ball and contact sports. This distribution of injury pattern is caused by the different sports’ biomechanics. Team sports with a higher proportion of physical contact (e.g., ice hockey, American football) and equitation have already been shown to account for a large proportion of SRCs in the past [2,26,27,28]. The mechanisms of concussion vary by type of sport and position, but do not necessarily require an impact to the head. For example, 5% of all concussions in American football, soccer and ice hockey are related to a trauma below the neck [29,30]. A longitudinal study determined a growth in the total number of concussions over the last decades. The evolution of professional athletes, who are better trained than in former times, and the development of sports to involve more speed, contact and risk has led to a higher risk of injuries to the head [31]. Moreover, recently developed protective gear can potentially lead to an increase in concussion rates by making athletes trust too much in the material and therefore take more risks during sports [32]. An important point of this investigation is the report of German data, which according to the epidemiology and types of sports is comparable to other European countries. Due to cultural specifics in treatment, the injury mechanism and epidemiology, the results have to be checked carefully before drawing worldwide conclusions.

Another interesting finding of this study was that women reported significantly more long-term symptoms than men after 6 months. It is known that women report more symptoms in acute trauma situations [33,34] and that these symptoms can last up to a month, especially in young patients [34].

In addition, the application of radiological imaging of the head with cCT and cMRI was investigated, according to the various types of sports and injury mechanisms. Overall, 41.1% of the patients had according radiological imaging. Odds ratio analysis showed at least a doubling of rates in cycling and equitation compared to ball sports. This was mainly conditioned by the injury mechanism, as in cycling and equitation, trauma was mostly caused by high-speed incidents and falls from a height. Due to trauma guidelines, radiological imaging should be processed in these cases [35,36]. The high rate of head imaging was also caused by the hierarchy of the study center. More severe cases are sent to the level 1 trauma departments, while milder injuries are more often treated in local hospitals [26,37]. However, in comparison to other studies, similar rates of sustained radiological imaging of the head have been observed [19].

When analyzing the behavior patterns and the first medical assessment directly after the injury, a lack of knowledge of how to act and behave was indicated by more than every second patient. In particular, in persons with—in their opinion—sufficient knowledge of concussions, as well as in athletes with insufficient knowledge, concussions were regarded as severe injuries. Nevertheless, 10.3% did not interrupt their training or match and 30.8% did not terminate physical activity directly after the incident. Although guidelines for red flag symptoms of concussions and different sideline screening tools exist, an integration of daily preclinical routine is rarely observed. Guidelines for sport-related concussions, for example, demand an immediate stop to the match or training and an interruption of the physical activity. In the next step, the affected athlete should be referred to a healthcare professional in order to be screened and treated [1,14,21,38,39]. However, many cases of concussions are still undetected, due to avoided medical assessment, and ignored or misunderstood symptoms. The introduction of a concussion law revealed this problem by noticing significantly more patients with concussion in emergency departments [40]. A good understanding of concussion, especially its symptoms and initial assessment, is a key factor in treatment and outcome. A lack of knowledge has been described among coaches, match officials and athletes and thus forms a basis for future prevention [41]. Bad management of SRC was not only found amongst athletes, but also in emergency departments. Rowe et al. found that one out of six patients in emergency departments were misdiagnosed despite, having clear symptoms of concussion [19]. Other investigations postulated bad management and lack of guidelines in hospitals, as well as insufficient realization [13,18,42]. Recently discovered correlation between biomarkers and concussions could help to reduce the number of misdiagnosed cases [21,43].

The importance of fundamental skills and information is reflected in the investigation of the first medical contact. In almost half of the incidents, other athletes (31.6%) or the patient himself (15.8%) were responsible for the initial assessment after trauma, as only a small number of patients had a medical professional as the initial contact. This turns out to be problematic, since interpreting the unspecific symptoms could result in symptoms being misunderstood or dismissed by nonprofessional consultation [19].

The perfect time to return to sport (RTS) after having an SRC is a highly discussed topic [15,29,42,44,45,46]. Each patient has to be treated individually by a step-by-step return to sport protocol. The Zurich statement, for example, recommends a return to moderate physical activity when the athlete is free of any symptoms, after an initial rest of 2 days without somatic or mental burden, and a return-to-competition after one week at the earliest [15]. In our study, the date of sport resumption was mainly set by the treating doctor in the hospital (73.3%), since there was no possibility for a secondary assessment of the patient after being released from the hospital. The median time of training absence was 14 days, but 28.6% of the patients did not comply with the determined timing of the return to sport. In a systematic review, Buettner et al. (2020) postulated a typical recovery time within 7 to 10 days after SRC [44]. In most recommendations, adequate rest is the main principle of treatment. Taking a break until concussion signs fade seems to be the best way to avoid the prolongation of symptoms so far [31,45], even if recent studies showed a converse thesis and demonstrated a reduced rate of PCS and a faster rehabilitation process after a shorter break, as well as an early beginning of moderate training [47,48]. Light aerobic exercise with cervical, vestibular, cognitive behavioral or vision therapy is also discussed as a potentially positive influence on returning to sport [46,49]. As the fading of symptoms can last longer or symptoms sometimes appear with a delay, the return to sport process should be surveilled by a physician [16,49]. Although coaches are more likely to refer to the physician’s advice, the majority of athletes want to decide on their own about the timing of the RTS. In particular, more ambitious and advanced athletes are known to force a faster return to training and competition activities, even if they take higher personal risks [17,21].

In our study, more than every second athlete already had experience of concussions in their past. The larger part had already been hospitalized for a concussion, and the majority had also been classified as exercise-related concussions. In some cases, the personal risk behavior of the concerned athletes seems to be associated with the appearance of SRC [31,50,51]. Another thesis for the repetitive cumulation of concussions among certain patients is based on a lack of regeneration after a previous SRC [31,52]. The pathophysiological correlate of this vulnerable phase is reduced cerebral blood flow, which was registered in athletes up to one month after concussion [22].

For future prevention, knowledge about sport-associated concussions should be increased [3,41], especially in athletes. For example, sideline evaluation tests (e.g., SCAT-5) are certified assessment tools for a fast evaluation of symptoms and should be more integrated in comprehensive usage [39,53,54,55,56]. As biomarker testing in the emergency department is still in the developmental process [43], medical personnel should be sensitized to SRC as well.

## 5. Conclusions

This retrospective investigation analyzed sports-related concussions in a level 1 emergency department in the course of a year. The study findings, such as the difference in the symptoms athletes complained to doctors about at the time of discharge from hospital in comparison to those they indicated in a questionnaire which was performed later on, or that long-term symptoms of SRC seem to significantly affect women more than men after 6 months, show that SRC is a difficult-to-treat disorder which requires a detailed, individually planned and patient-involving treatment process.

## Figures and Tables

**Table 1:** Distribution of sports-related concussions (SRC) by type of sport and injury mechanism, *n* = 85.

Type of Sport	Impact through Direct Physical Contact	Impact through Ball or Other Object	Fall with Subsequent Impact without Contact	Fall with Subsequent Impact with Contact	Other
Soccer	13 *	5	4	-	-
Cycling sport	-	-	9 *	4	-
Equitation	-	-	22 *	-	2
Mountain Biking	-	1	3	-	1
Winter Sports	-	-	3	-	1
Climbing	-	-	5	-	-
Swimming	1	-	-	-	-
Martial arts	-	1	-	-	-
Paragliding	-	-	1	-	-
Running	-	-	1	-	-
Other ball sports	5 *	1	2	-	-

* *p* < 0.05.

**Table 2:** Medical record information on sports-related concussion.

Hospitalization in Days	% (*n* = 85)
no hospitalization	5.9 (5)
6 h	1.2 (1)
24 h	75.3 (64)
48 h	8.2 (7)
72 h	2.4 (2)
4 days or longer	7.0 (6)
Main symptoms after concussion	% (*n* = 85)
unconsciousness < 30 min	41.2 (35)
headache	36.5 (31)
retrograde amnesia	29.4 (25)
nausea	23.5 (20)
vertigo	21.2 (18)
vomiting	11.8 (10)
Symptom duration	% (*n* = 38)
1 to 4 h	42.1 (16)
4 to 12 h	13.2 (5)
12 to 24 h	5.3 (2)
24 to 72 h	10.5 (4)
3 to 7 days	21.1 (8)
more than 7 days	7.9 (3)
Pretrauma existing neuropsychiatric disorders	% (*n* = 39)
chronic headache or migraine	33.3 (13)
ADHD, dyslexia	7.7 (3)
depression or any other psychiatric disease	12.8 (5)
one of these disorders in family	25.6 (10)
First assessment after trauma	% (*n* = 38)
medical personnel	18.4 (7)
physiotherapist	7.9 (3)
coach	10.5 (4)
patient himself/herself	15.8 (6)
teammate	31.6 (12)
others	15.8 (6)

ADHD: attention deficit hyperactivity disorder.

**Table 3:** Return to sports information of the athletes with a sports-related concussion.

Schedule for Return to Sports (RTS)	% (*n* = 39)
yes	48.7 (19)
no	48.7 (19)
not known	2.6 (1)
Person who set the schedule	% (*n* = 15)
doctor in hospital	73.3 (11)
general doctor	13.3 (2)
athlete himself/herself	13.3 (2)
Adherence to deadline	% (*n* = 21)
yes	71.4 (15)
no	28.6 (4)
Successful return to sport	% (*n* = 39)
yes	89.7 (35)
no	10.3 (4)

## Data Availability

The dataset is available on request.
